# Beyond ‘test and treat’ – malaria diagnosis for improved pediatric fever management in sub-Saharan Africa

**DOI:** 10.3402/gha.v9.31744

**Published:** 2016-12-16

**Authors:** Emily White Johansson

**Affiliations:** Department of Women's and Children's Health, International Maternal and Child Health, Akademiska sjukhuset, Uppsala University, Uppsala, Sweden

**Keywords:** malaria, IMCI, child health, diagnosis, fever case management

## Abstract

**Background:**

Malaria rapid diagnostic tests (RDTs) have great potential to improve quality care and rational drug use in malaria-endemic settings although studies have shown common RDT non-compliance. Yet, evidence has largely been derived from limited hospital settings in few countries. This article reviews a PhD thesis that analyzed national surveys from multiple sub-Saharan African countries to generate large-scale evidence of malaria diagnosis practices and its determinants across different contexts.

**Design:**

A mixed-methods approach was used across four studies that included quantitative analysis of national household and facility surveys conducted in multiple sub-Saharan African countries at the outset of new guidelines (Demographic and Health Surveys and Service Provision Assessments). Qualitative methods were used to explore reasons for quantitative findings in select settings.

**Results:**

There was low (17%) and inequitable test uptake across 13 countries in 2009–2011/12, with greater testing at hospitals than at peripheral clinics (odds ratio [OR]: 0.62, 95% confidence interval [CI]: 0.56–0.69) or community health workers (OR: 0.31, 95% CI: 0.23–0.43) (Study I). Significant variation was found in the effect of diagnosis on antimalarial use at the population level across countries (Uganda OR: 0.84, 95% CI: 0.66–1.06; Mozambique OR: 3.54, 95% CI: 2.33–5.39) (Study II). A Malawi national facility census indicated common compliance to malaria treatment guidelines (85% clients with RDT-confirmed malaria prescribed first-line treatment), although other fever assessments were not often conducted and there was poor antibiotic targeting (59% clients inappropriately prescribed antibiotics). RDT-negative patients had 16.8 (95% CI: 8.6–32.7) times higher odds of antibiotic overtreatment than RDT-positive patients conditioned by cough or difficult breathing complaints (Study III). In Mbarara (Uganda), health workers reportedly prescribed antimalarials to RDT-negative patients if no other fever cause was identified and non-compliance seemed further driven by RDT perceptions, system constraints, and client interactions (Study IV).

**Conclusions:**

A shift from malaria-focused *test and treat* strategies toward *IMCI with testing* is needed to improve quality care and rational use of both antimalarial and antibiotic medicines. Strengthened health systems are also needed to support quality clinical care, including adherence to malaria test results, and RDT deployment should be viewed as a unique opportunity to contribute to these important efforts.

## Introduction

Approximately 6 million children less than 5 years of age die annually worldwide, and half of these deaths occur in sub-Saharan Africa (1). These deaths are largely caused by complications during the newborn period or common infections that are both preventable and treatable, notably pneumonia, malaria, and diarrhea (2). A cornerstone of child survival programs, therefore, includes expanding access to early and effective treatment for these conditions, particularly to reach the poorest children at highest risk of death (3).

### Evolution of treatment guidance for childhood illnesses

Efforts to effectively treat these conditions have historically been hampered by limited diagnostics available at peripheral clinics in order to identify the underlying cause of disease. Patients in these contexts are often treated on the basis of symptom presentations and simple clinical assessments, which, for many years, was guided by disease-specific protocols that did not account for comorbidities or symptom overlaps across disease conditions (4). This led to a growing recognition during the 1980s and the 1990s that integrated treatment protocols are essential for more appropriate and cost-effective management of the sick children.

Since the 1990s, the World Health Organization (WHO) and United Nations Children's Fund (UNICEF) have promoted the Integrated Management of Childhood Illness (IMCI) guidelines, which provide an algorithm to help clinically manage the most common causes of child morbidity and mortality in an integrated manner (5). IMCI algorithms target the following common childhood conditions: malaria, pneumonia, diarrhea, dehydration, measles, malnutrition, anemia, and ear problems, among others. These guidelines also indicate danger signs of severe diseases that need immediate referral.

Fever is a common symptom of many of the abovementioned conditions. Yet, IMCI has historically recommended presumptive malaria treatment of pediatric fevers in malaria-endemic settings, given high malaria mortality rates, quick progression from clinical symptoms to death, and a lack of other defining features for clinical differentiation. Importantly, the presumption of all fevers as ‘malaria’ has been shown to impede probing for other causes of fever, notably pneumonia, which is also a leading cause of child deaths (6). Indeed, malaria and pneumonia have substantial symptom overlaps such that nearly all sick children with IMCI-classified pneumonia (cough with a breathing rate of 50 or more breaths per minute in children aged 2 to 12 months or 40 or more breaths per minute in children aged 12 months to 5 years) also had reported fever, or IMCI-classified malaria (7). Although these children should receive dual classifications and treatments for both conditions, studies have shown common mistreatment of IMCI-classified pneumonia with antimalarials, which delays appropriate care with potentially fatal consequences (8).

### From clinical management to diagnostic confirmation of malaria

In 2010, the WHO revised malaria treatment guidelines to promote universal malaria diagnosis for all patients in all transmission settings with treatment based on the test result. This shift in guidelines was based on expert guidance and increasing availability of malaria rapid diagnostic tests (RDTs) that make diagnosis possible in peripheral clinics that previously lacked this capacity (9, 10). Malaria RDTs satisfy established criteria for assessing point-of-care diagnostic tests in terms of being affordable, sensitive, specific, user-friendly, robust, equipment-free, and deliverable to locations where diagnosis is most needed (11).

Many other reasons also supported the shift toward test-based malaria case management, notably the need to improve quality care for other fever causes as previously described (9). There has also been growing concerns about antimalarial drug resistance that is exacerbated by inappropriate prescriptions and poor adherence practices (12). Rising antimalarial drug costs also drive the need for better precision in treatment (13), and improved treatment targeting is particularly important in settings that significantly reduced malaria transmission in recent years where a lower fraction of fevers is now due to malaria (14). Finally, malaria surveillance has historically been hindered by imprecise case definitions based on reported fever symptoms such that recording only confirmed cases could improve malaria monitoring and burden estimation (9).

National programs have greatly invested in large-scale RDT deployment in recent years with the expectation of achieving these objectives (15). Yet, research from various settings has shown mixed program success (16). While most research generally indicates reduced antimalarial prescriptions after RDT introduction into health facilities, many adherence studies found common prescribing of antimalarial drugs to RDT-negative patients (16). In studies where first-line malaria treatment (artemisinin-based combination therapies [ACT]) was largely restricted to positive cases, some research further reported widespread prescriptions of second-line antimalarial or antibiotic medicines to negative test results and not according to established clinical guidelines (17, 18).

### Thesis rationale and objectives

This evidence has largely been derived from small-scale adherence studies conducted in a limited set of countries, particularly Kenya, Malawi, Tanzania, Uganda, and Zambia (16). Although evidence has grown since the start of this doctoral project, there remains limited information for many countries that have comparably invested in RDT deployment (15, 16). Although a few countries have conducted national surveys to examine malaria testing practices across facility contexts (19, 20), there is still limited understanding of contextual differences and factors associated with RDT use and adherence. In addition, few facility studies have examined RDT integration into established clinical practice, or IMCI for sick children, to improve overall quality care for febrile children. Finally, and importantly, pediatric fevers in these settings are commonly managed at informal sources within communities, and initial RDT deployment targeted only formal health system sources (21). This could potentially result in low testing rates among the general population, which had not been adequately documented given the primary focus on facility-based research.

The PhD thesis emerged from the identification of these critical evidence gaps coupled with an awareness of new malaria diagnosis data collection in nationally representative population- and facility-based surveys routinely implemented across low- and middle-income countries (Demographic and Health Surveys [DHS], Malaria Indicator Surveys [MIS] and Service Provision Assessments [SPA]) (22, 23). The PhD thesis aimed to use these new data to answer some of these important research questions and to combine with qualitative research to better understand reasons for observed practices. Specific research questions included are as follows:

- What are the extent and determinants of malaria diagnostic test use for pediatric fevers in 13 sub-Saharan African countries? How do malaria risk and the source of care influence test uptake? (Study I)
- Does malaria testing reduce antimalarial drug use and increase antibiotic use for pediatric fevers at the population level? Are there effect differences across countries and what are the reasons for variation? (Study II)
- How are sick children with fever complaints assessed and treated using RDT and IMCI during outpatient consultations in Malawi health facilities? What factors are associated with poor treatment decisions for clients with fever symptoms (e.g. antibiotic or antimalarial overtreatment)? (Study III)
- How do health workers manage RDT-negative febrile children in the low-risk areas of Mbarara District (Uganda)? What are perceived challenges in this work and what are some desires for additional support or tools to help manage these cases? (Study IV)

### Conceptual framework

**Fig. 1:**
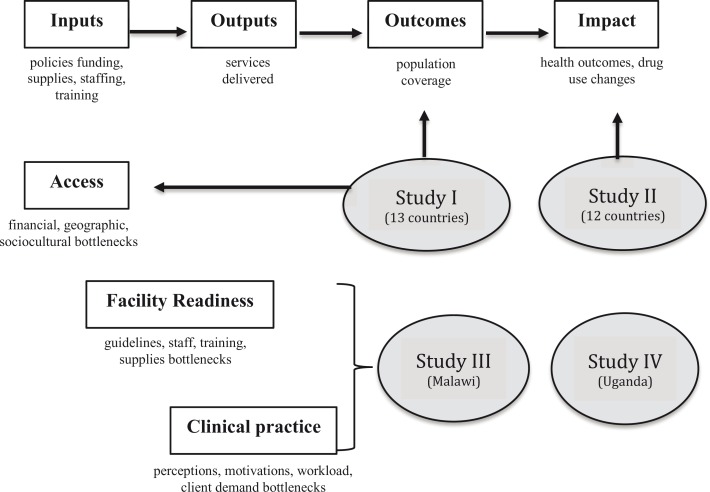
Conceptual framework.

The conceptual framework for the PhD thesis embedded a bottleneck analysis into the standard monitoring and evaluation (M&E) framework for assessing health system performance (24, 25) (Fig. 1). The bottleneck analysis highlights underlying factors at intermediate stages of service delivery – *access*, *facility readiness*, and *clinical practice* – that could impede achievement of expected outcomes and impact defined in the M&E framework.

*Access* bottlenecks refer to geographic, financial, or socioeconomic barriers to using health services where malaria diagnosis is available. *Facility readiness* bottlenecks refer to facility or system constraints, such as guidelines, staffing, training, and referral systems, for using malaria diagnosis to improve pediatric fever management. *Clinical practice* bottlenecks refer to issues during the clinical encounter, such as perceptions, motivations, and client interactions, that could impede employing malaria diagnosis as intended by guidelines.

Some advantages of this modification for the PhD thesis included capturing *access* bottlenecks that are not explicitly measured in the standard M&E framework. In addition, the standard M&E framework quantitatively measures inputs to a system and their resulting outputs, outcomes, or impact. Yet, impediments to effective service delivery may reflect underlying perceptions or motivations that are not easily quantified and better captured using qualitative methods as part of a bottleneck analysis.

## Methods

A mixed-methods approach was used across the four studies in the PhD thesis. Table 1 summarizes the different methodologies used across the four studies that are further described in the published articles (26–29).

**Table 1:** Methods overview

Study	Design	Data source	Sample size	Methods	Outcomes, main predictors, and themes
I	Meta-analysis 13 national cross-sectional population-based surveys	DHS and MIS in 13 countries in 2009–2011/12	27,916 febrile children under 5 years in 13 countries (2009–2011/12)	Mixed-effects logistic regression models in the pooled data set for 13 countries	Outcome(s): Malaria diagnostic test uptake Main predictor(s): Malaria risk and source of care
II	Mixed-methods Quantitative: national cross-sectional population-based surveys Qualitative: Country case studies	Quantitative DHS in 12 countries 2010–2012 Qualitative: Expert consultations; document reviews; published research	Quantitative 16,323 febrile children under 5 years and taken to care in 12 countries (2010–2012) Qualitative: six country case studies	Quantitative Mixed-effects logistic regression models in individual country datasets Qualitative: Multiple case studies leading to a cross-case synthesis. Thematic analysis identified recurring themes	Quantitative Outcome(s): ACT use, any antimalarial use, any antibiotic use Main predictor(s): Malaria test uptake Qualitative (themes): RDT deployment; health system structure; malaria epidemiology; RDT practice perceptions
III	Data mining national facility census	Malawi SPA 2013–2014	977 audited facilities; 2,950 observed sick child clients 2–59 months in first visit for illness	Classification trees using model-based recursive partitioning	Outcome(s): Antibiotic and antimalarial overtreatment Main predictor(s): RDT results
IV	Qualitative	In-depth interviews and FGDs in Mbarara District, Uganda	20 health worker interviews; 7 caregiver focus group discussions	Latent content analysis	Qualitative (themes) RDT perceptions, strategies to differentiate fever causes, desires for other tools or support, perceived challenges

DHS, Demographic and Health Surveys; MIS, Malaria Indicator Surveys; ACT, artemisinin-based combination therapies; RDT, rapid diagnostic tests; FGD, focus group discussions; SPA, service provision assessments.

### Data sources

Three (of four) PhD studies analyzed the DHS, MIS, or SPA that are nationally representative population- and facility-based cross-sectional surveys routinely implemented in low- and middle-income countries. These survey programs and their methodologies are described elsewhere (22, 23). The fourth PhD paper was a qualitative study conducted in Mbarara District, Uganda, as described below.

### Study settings

**Fig. 2:**
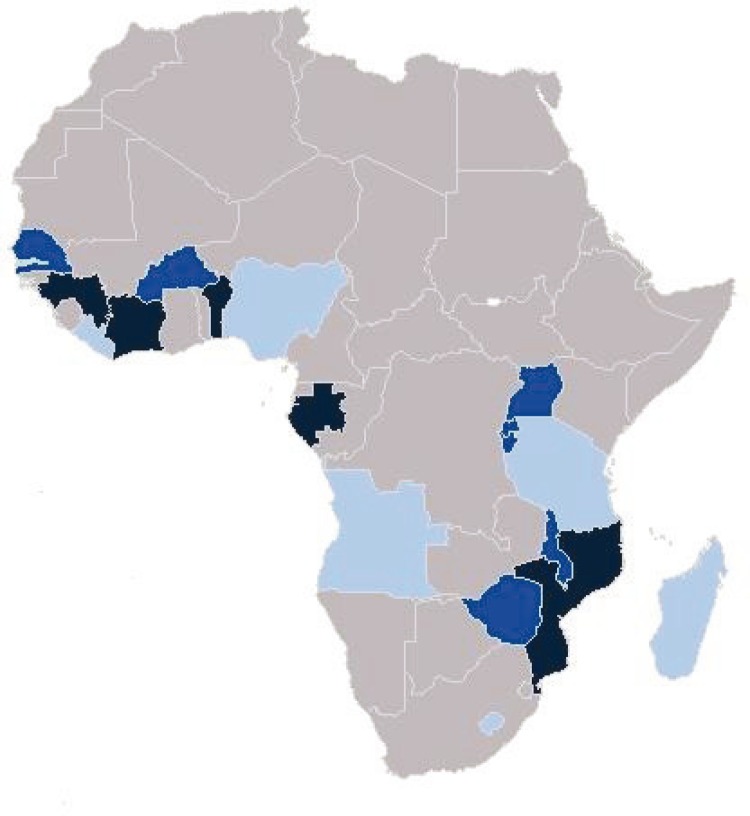
Map of countries. Dark blue (Study I countries); light blue (Study II countries); medium blue (Studies I and II countries). **Study I** included Angola (MIS 2011), Burkina Faso (DHS 2010–2011), Burundi (DHS 2010–2011), Lesotho (DHS 2009–2010), Liberia (MIS 2011), Madagascar (MIS 2011), Malawi (DHS 2010), Nigeria (MIS 2010), Rwanda (DHS 2010–2011), Senegal (DHS 2010–2011), Tanzania (AIS/MIS 2011–2012), Uganda (DHS 2011), and Zimbabwe (DHS 2010–2011). **Study II** included Benin (DHS 2011–2012), Burkina Faso (DHS 2010–2011), Burundi (DHS 2010–2011), Cote d'Ivoire (DHS 2011–2012), Gabon (DHS 2012), Guinea (DHS 2012), Malawi (DHS 2010), Mozambique (DHS 2011), Rwanda (DHS 2010–2011), Senegal (DHS 2010–2011), Uganda (DHS 2011), and Zimbabwe (DHS 2010–2011). **Study III** analyzed a Malawi national facility census (2013–2014). **Study IV** was a qualitative study in Uganda (Mbarara District).

Study I included 13 sub-Saharan African countries using DHS and MIS conducted in 2009–2011/12. Study II was based on 12 sub-Saharan African countries using DHS conducted in 2010–2012. Study III analyzed the Malawi national facility census from 2013 to 2014 that included both public and private sources. Study IV was a qualitative study conducted in Mbarara District that included in-depth interviews with health workers at peripheral clinics and focus group discussions (FGD) with caregivers living in facility catchment areas. Mbarara District has low malaria prevalence (30) and is located 270 km southwest of the capital city. Mbarara is mostly a rural area with subsistence agriculture that has grown in recent years due to its central location as a transport hub to neighboring countries. The district includes 58 health facilities, both public (49) and private (9), as well as community health workers and private drugs shops that are commonly used to obtain medicines (31, 32). Figure 2 shows the countries included in these different studies.

### Malaria diagnosis definitions

In DHS and MIS used in Studies I–II, malaria diagnosis receipt was based on caregiver responses that his or her child less than 5 years old with a reported fever episode in the previous 2 weeks received a finger or heel stick for testing, which refers to RDT or microscopy for malaria diagnosis. In SPA used in Study III, malaria diagnostic test use derived from provider reports that an RDT was performed before or during the consultation, and if so, the RDT result was subsequently reported. Referral for malaria diagnosis is based on caregiver reports during the exit interview that the provider instructed him or her to take the child to another provider or to go to the laboratory in this facility for a finger or heel stick for blood testing.

### Treatment definitions

In DHS used in Study II, treatment outcomes included ACT use, any antimalarial use, and any antibiotic use. These treatment outcomes were based on reported drugs taken by children under age 5 with a febrile illness in the previous 2 weeks. In SPA used in Study III, antibiotic overtreatment was the main outcome, which was defined as any antibiotic prescription ‘without antibiotic need’ or excluding clients with IMCI pneumonia based on re-examination (see definition in the Introduction section) and additionally excluding clients diagnosed with the following conditions during the consultation: sepsis, dysentery, acute ear infection, mastoiditis, abscess, or severe malnutrition.

### Data analysis

Study I was a meta-analysis of 13 DHS and MIS using mixed-effects logistic regression models to quantify the influence of source of care, malaria risk, and other socioeconomic variables on malaria diagnostic test use for pediatric fevers in combined and individual country data sets (Table 1).

Study II analyzed individual country data sets from 12 DHS using mixed-effects logistic regression models to separately quantify the influence of malaria diagnostic test use for pediatric fevers on three binary outcomes: ACT use, any antimalarial use, and any antibiotic use. A multiple case study design was employed to plausibly explain unexpected findings from six countries, which led to a cross-case synthesis to identify common themes that could explain contrasting country results (33) (Table 1).

Study III analyzed a Malawi national facility census to assess integrated pediatric fever management among children aged 2 months to 5 years in terms of other complaints, assessments completed and drug prescriptions for RDT-confirmed malaria, IMCI pneumonia, and clinical diarrhea. Classification trees using model-based recursive partitioning analyzed the effect of RDT results on antibiotic overtreatment and learned the influence of 38 other partitioning variables on this relationship at the patient, provider, and facility levels (34, 35). Classification trees are well-suited for this analysis since there may be numerous influences on the examined relationships of RDT results and antibiotic over-treatment, including potentially complex interactions among these 38 variables that are not easily detected using standard statistical methods and where there is no clear hypothesis about these sub-group interactions for statistical testing. Model-based recursive partitioning, in particular, embeds a parametric model into a recursive partitioning algorithm used in classification trees in order to automatically detect subgroup interactions with the binary outcome of antibiotic overtreatment. This is carried out by initially fitting a crude mixed-effects logistic regression model to the full data set and specifying the 38 input variables for repeated testing in order to partition the data set into subgroups where there may be different patterns of association between the main exposure and outcome. Parameter instability is repeatedly assessed across these partitioning variables and the data set is partitioned into nodes (or subgroups) according to the variable causing the highest instability. This automatic process is repeated at each node until no additional significant classifiers are identified using a Bonferroni-adjusted significance level of 0.05 or the minimal node size of 20 observations is reached. The parametric model is refit to each identified node yielding a series of segmented parametric models, including odds ratios or other coefficients that estimate the association between the main exposure and the outcome across each identified node or subgroup (Table 1).

Study IV was a qualitative study in Mbarara District, which included 20 health worker interviews at lower level clinics and seven FGD with caregivers living in facility catchment areas. Interviews were based on a semi-structured guide to capture how non-malaria pediatric fevers are currently managed in this district, perceived challenges in this work, and desires for additional tools or support. Caregivers were asked about their RDT experiences, perceptions of pediatric fever causes, and the acceptability of withholding antimalarials for RDT-negative cases. A latent content analysis approach was used to analyze interviews and FGD transcripts (36) (Table 1).

## Results

Thesis findings are summarized according to bottleneck stages outlined in the conceptual framework that could hamper the effective use of malaria diagnosis for improved pediatric fever management: *access, facility readiness*, and *clinical practice*.

### Access

Malaria diagnostic test use for pediatric fevers was low (17%) and inequitable across 13 countries in 2009–2011/12. This analysis included 105,791 children under 5 years of age, of which 27% had a reported fever in the previous 2 weeks. Table 2 highlights that seeking care from peripheral or informal sources significantly reduced the odds of malaria test receipt, which indicates an important *access bottleneck*. There was significantly lower testing odds if the febrile children were taken to a non-hospital formal source (odds ratio [OR]: 0.62, 95% confidence interval [CI]: 0.56–0.69) or community health worker (OR: 0.31, 95% CI: 0.23–0.43) compared to hospital attendance. Other important socioeconomic inequities were also identified, which further suggest this *access* bottleneck. Children with fever living in wealthiest households had higher testing odds than those in poorest households (OR 1.63: 95% CI: 1.39–1.91), and febrile children with mothers having at least secondary education had higher testing odds than those with mothers who had no formal education (OR: 1.33, 95% CI: 1.16–1.54). Living in high-risk areas also reduced the odds of testing compared with living in low risk areas (OR: 0.51, 95% CI: 0.42–0.62).

**Table 2:** Effect of source of care, malaria endemicity, and socioeconomic variables on test uptake in 13 studied countries in 2009–2011/12

		Adjusted OR	(95% CI)	*p value*
Source of care	Hospital	1.00		
	Non-hospital formal medical	0.62	(0.56–0.69)	<0.001
	Community health worker	0.31	(0.23–0.43)	<0.001
	Pharmacy	0.06	(0.05–0.09)	<0.001
	Other	0.10	(0.08–0.13)	<0.001
	No care sought	0.05	(0.04–0.06)	<0.001
Malaria endemicity	No transmission	0.46	(0.34–0.63)	<0.001
	Unstable	1.32	(0.11–15.50)	0.823
	Low stable	1.00		
	Moderate stable	1.04	(0.86–1.25)	0.697
	High stable	0.51	(0.42–0.62)	<0.001
Child's age (in months)	0–5	0.72	(0.59–0.87)	0.001
	6–11	1.00		
	12–23	1.24	(1.09–1.41)	0.001
	24–35	1.27	(1.11–1.45)	<0.001
	36–47	1.10	(0.95–1.26)	0.203
	48–59	1.18	(1.02–1.37)	0.030
Child's sex	Male	1.00		
	Female	0.98	(0.91–1.06)	0.676
Maternal age (in years)	15–24	1.00		
	25–29	1.01	(0.91–1.12)	0.891
	30–34	1.06	(0.94–1.20)	0.336
	35– 39	1.06	(0.92–1.21)	0.425
	40–49	0.99	(0.83–1.17)	0.890
Maternal education	No attendance	1.00		
	Primary	1.32	(1.19–1.46)	<0.001
	Secondary or higher	1.33	(1.16–1.54)	<0.001
Household wealth	Poorest	1.00		
	Second	0.99	(0.87–1.13)	0.850
	Middle	1.03	(0.90–1.18)	0.670
	Fourth	1.21	(1.06–1.40)	0.006
	Least poor	1.63	(1.39–1.91)	<0.001
Household members	0–4 members	1.00		
	5–8 members	0.95	(0.86–1.05)	0.307
	9–12 members	0.87	(0.76–0.99)	0.036
	13 or more members	0.66	(0.54–0.80)	<0.001
Residence	Urban	1.00		
	Rural	0.71	(0.62–0.82)	<0.001

Mixed-effects logistic regression model in pooled data set of 13 surveys, adjusted for data clustering and above covariates. Unstable malaria transmission refers to areas of very low but non-zero transmission. Stable transmission categories refer to low (*Pf*PR_2–10_ <5%), moderate (*Pf*PR_2–10_ 5–40%), and high (*Pf*PR_2–10_>40%). CI, confidence interval; AOR, adjusted odds ratio.

The *access bottleneck* was further identified in the study that related malaria diagnosis to changing drug use patterns for pediatric fevers at the population level. These results indicated that no country reduced ACT use associated with testing as hypothesized, and significant country variability was found in the impact of testing on pediatric fever treatment (Uganda OR: 0.84, 95% CI: 0.66–1.06; Mozambique OR: 3.54, 95% CI: 2.33–5.39). Four common themes explained varying findings across countries: available diagnostics and medicines, quality of care, care-seeking behavior, and malaria epidemiology. Available diagnostics and medicines are a theme relating to *access* bottlenecks and were a central explanation for results across country case studies. Indeed, children getting tested were probably at locations that also had medicines. In some countries, diagnostic services were only available at hospitals that likely also had better drug availability and may have been attended by patients with more severe illnesses. Taken together, these factors could greatly increase the treatment likelihood in these countries, which further indicates an important *access* bottleneck.

### Facility readiness and clinical practice

*Facility readiness* and *clinical practice* bottlenecks are often intertwined and are therefore explored together in this section by presenting results separately from quantitative and qualitative studies in Malawi and Uganda, respectively.

#### Integrated pediatric fever management in Malawi health facilities

Integrated pediatric fever management using RDT and IMCI was examined in sick child observations of a national facility census conducted in Malawi in 2013–2014. This census included 977 facilities out of 1,060 on the Ministry of Health master list. A total of 2,950 sick children aged 2–59 months visiting for a first-time illness were observed and 1,981 had reported fever complaints during the exit interview. Among these 1,981 clients with fever, 1,436 (72%) reported cough or difficult breathing (CDB) complaints, 569 (29%) reported diarrhea complaints, and 359 (18%) reported other issues such as skin, eye, and ear problems. Any danger sign was reported among 1,021 clients (52%) with fever complaints. Only 117 (6%) reported fever alone with no other complaints or danger signs.

Among 1,981 febrile clients, 1,426 (72%) had RDT performed or were referred for malaria diagnosis, 44 (2%) had their necks checked for stiffness, 524 (27%) had palms checked for pallor, 185 (9%) were checked for mouth disorders, and 563 (28%) were undressed for examination. Among 1,436 clients with both fever and CDB complaints, 256 (18%) had breathing rates counted for 60 s (Table 3).

**Table 3:** Completed assessments of clients with fever complaints, Malawi health facilities, 2013–2014

	*N*	% assessed (95% CI)
Fever complaint	1,981	
Fever mentioned or asked about by provider	1,684	85.0 (82.8–87.2)
Temperature taken or body felt for hotness	1,386	70.0 (65.5–74.1)
RDT done prior to consultation or referral for malaria diagnosis	1,426	72.0 (69.0–74.7)
Checked neck for stiffness	44	2.2 (1.4–3.5)
Checked for pallor by looking at palms	524	26.5 (23.5–29.6)
Looked into child's mouth	185	9.3 (7.4–11.6)
Undressed child to examine (up to shoulders or down to ankles)	563	28.4 (25.2–31.9)
Fever and CDB complaint	1,436	
Both symptoms mentioned or asked about by provider	1,010	70.3 (66.7–73.7)
Counted breaths for 60 s	256	17.8 (14.8–21.2)
Fever and diarrhea complaint	569	
Both symptoms mentioned or asked about by provider	307	53.9 (48.3–59.4)
Checked skin turgor for dehydration	98	17.3 (13.3–22.1)

Symptom complaints are based on caregiver reports during exit interviews. Completed assessments are based on recorded observations during consultations. Point estimates are weighed to account for unequal probabilities of selection due to differing client volumes on the interview date. Standard error estimation accounted for clustering of client observations within facilities. CI, confidence interval; RDT, rapid diagnostic tests; CDB, cough or difficult breathing.

Tables 4 and 5 show that 312 febrile clients had a reported RDT-positive result, and 265 (85%) with RDT-confirmed malaria were prescribed first-line antimalarial treatment, while 25 (8%) received no antimalarial prescription (antimalarial undertreatment). Among 434 with reported RDT-negative results, 44 (10%) were prescribed any antimalarial drug (antimalarial overtreatment). Among 376 with IMCI pneumonia based on re-examination, 148 (39%) received first-line antibiotic treatment and 105 (28%) received no antibiotic prescription (antibiotic undertreatment). A total of 1,411 clients were categorized as ‘without antibiotic need’, who did not have IMCI pneumonia based on re-examination and were not given the following diagnoses during the consultation: sepsis, acute ear infection, mastoiditis, dysentery, abscess, and severe malnutrition. Among these clients ‘without antibiotic need’, 830 (59%) were prescribed any antibiotic drug (antibiotic overtreatment).

**Table 4:** Antimalarial prescriptions for clients with fever complaints, Malawi health facilities, 2013–2014

	*N*	% with prescription (95% CI)
Fever complaint	1,981	
RDT done prior to consultation or malaria diagnosis referral	1,426	
RDT done prior to consultation with result reported	746	
RDT-positive result	312	
First-line antimalarial prescription	265	85.1 (77.5–90.4)
Second-line antimalarial prescription	22	7.0 (4.4–10.8)
No antimalarial prescription	25	7.9 (3.6–16.7)
RDT-negative result	434	
Any anti-malarial prescription (overtreatment)	44	10.2 (6.8–14.9)

First-line antimalarial prescription is defined as artemether or artesunate (oral, injection, or suppository) or ACT/AL (oral) prescription for an RDT-positive result. Second-line antimalarial prescription is defined as quinine (oral or injection), amodiaquine (oral), fansidar (oral), or other antimalarial (oral or injection) prescription for an RDT-positive result. Antimalarial undertreatment is defined as no antimalarial prescription for an RDT-positive result. Anti-malarial overtreatment is defined as any antimalarial prescription for an RDT-negative result. Point estimates are weighed to account for unequal probabilities of selection due to differing client volumes on the interview date. Standard error estimation accounted for clustering of client observations within facilities. CI, confidence interval; RDT, rapid diagnostic tests.

**Table 5:** Antibiotic prescriptions for clients with fever complaints, Malawi health facilities, 2013–2014

	*N*	% with prescription (95% CI)
Fever complaint	1,981	
IMCI pneumonia assessment with result reported	1,367	
IMCI pneumonia positive classification	376	
First-line antibiotic prescription	148	39.4 (32.3–46.9)
Second-line antibiotic prescription	123	32.7 (26.3–39.8)
No antibiotic prescription	105	27.9 (20.7–36.5)
‘Without antibiotic need’	1,411	
Any antibiotic prescription (overtreatment)	830	58.8 (55.1–62.4)

First-line antibiotic prescription is defined as benzyl penicillin injection or amoxicillin (capsules or syrup) for IMCI pneumonia. Second-line antibiotic prescription is defined as cotrimoxazole (syrup or tablets) or other antibiotics (injection, syrup, or capsule) for IMCI pneumonia. Antibiotic undertreatment is defined as no antibiotic prescription for IMCI pneumonia. Antibiotic overtreatment is defined as any antibiotic prescription ‘without antibiotic need’, or excluding clients with IMCI pneumonia (based on re-examination) and additionally excluding clients with the following diagnostic categories (recorded in the consultation): sepsis, dysentery, mastoiditis, acute ear infection, abscess, or severe malnutrition. Point estimates are weighed to account for unequal probabilities of selection due to differing client volumes on the interview date. Standard error estimation accounted for clustering of client observations within facilities. CI, confidence interval; IMCI, Integrated Management of Childhood Illness.

**Fig. 3:**
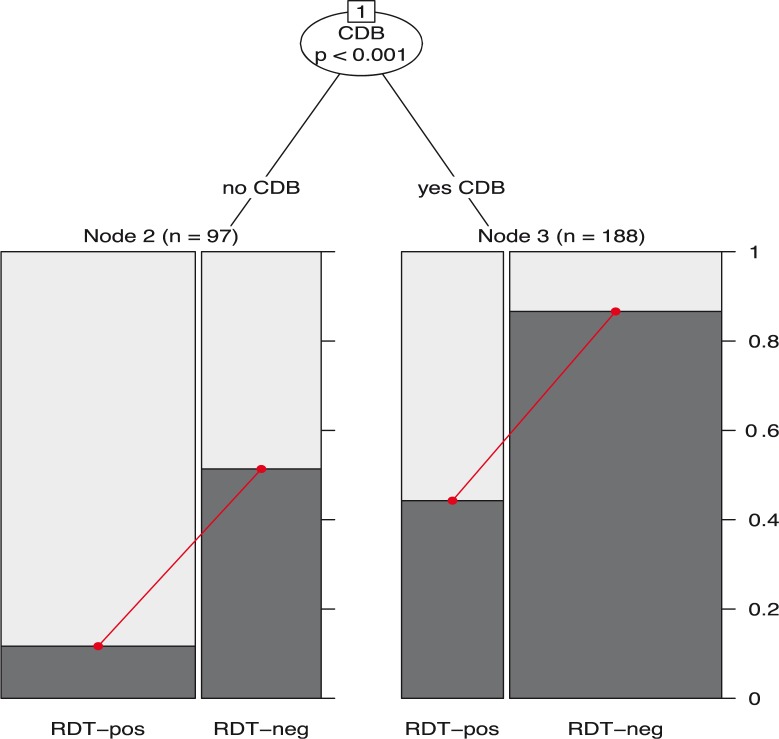
Interrelationship between RDT results and other variables on antibiotic overtreatment among clients with fever complaints, Malawi health facilities, 2013–2014. Red lines indicate the effect size of RDT results on antibiotic overtreatment in each subgroup. Dark gray bars indicate any antibiotic prescription among clients ‘without antibiotic need’ referred to as antibiotic overtreatment. CDB refers to cough or difficult breathing complaint. AB refers to antibiotic. Results are derived from a model-based recursive partitioning approach that initially fit a mixed-effects logistic regression model to estimate the relationship between the RDT result and antibiotic overtreatment, and the influence of 38 other partitioning variables at patient, provider, and facility levels was learned through recursive partitioning conditional on the main predictor included in the model.

An analysis of influences on antibiotic overtreatment was conducted among 526 clients ‘without antibiotic need’ and with a reported RDT result using a model-based recursive partitioning approach. A crude mixed-effects logistic regression model was initially fit to the data set that indicated 16.8 (95% CI: 8.6–32.7) times higher odds of antibiotic overtreatment among RDT-negative clients than positive cases. Fig. 3 depicts all clients ‘without antibiotic need’, and the dark gray bars indicate those receiving any antibiotic drug or antibiotic overtreatment. The figure shows that CDB complaint was a statistically significant classifier learned through recursive partitioning. This split was largely driven by a difference in the underlying risk of antibiotic overtreatment across clients with or without CDB complaints rather than a difference in effect size across groups. Clients without CDB complaints and an RDT-positive result had very low risk of antibiotic overtreatment, and this risk increased with a negative test result. A similar pattern was found among clients with CDB complaints, although there was greater underlying risk of antibiotic overtreatment in this group. The highest risk of antibiotic overtreatment was found among clients with CDB complaints and an RDT-negative result. In this group, 82% of clients with RDT-negative results and CDB complaints were inappropriately prescribed antibiotics according to the study definition.

#### RDT perceptions and experiences in Mbarara District (Uganda)

**Fig. 4:**
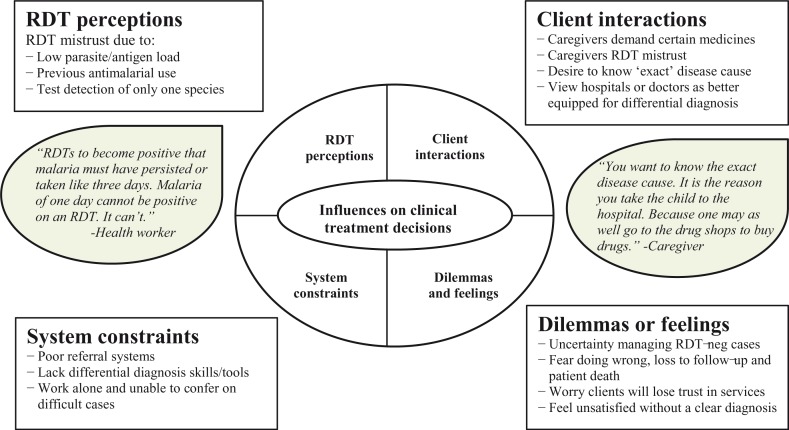
Qualitative themes describing influences on managing non-malaria pediatric fevers in Mbarara District, Uganda.

In Mbarara District, health workers described various perceptions and challenges to using RDT for improved pediatric fever management, which highlight important *facility readiness* and *clinical practice* bottlenecks. Qualitative findings suggested that antimalarial medicines were commonly prescribed to RDT-negative results in this low transmission area if no alternative fever cause could be found. Malaria overtreatment also seemed driven by a combination of RDT perceptions, provider–client interactions, and system constraints that, in turn, elicited various dilemmas or feelings in health workers (Fig. 4).

RDT perceptions included a general RDT mistrust that was fueled by experiences of multiple testing, giving contradictory results (e.g. RDT negative but blood smear positive). This mistrust was also driven by expectations of false negative results in specific scenarios: low parasite or antigen loads, taking a recent antimalarial dose, or RDT detection of *Plasmodium falciparum* only. First, many health workers explained it would be difficult for a patient to receive a positive RDT result during early clinical symptoms (up to 3 days from fever onset) due to low parasite or antigen loads. Second, health workers talked about prescribing malaria treatment even if the RDTs were negative if the patient had recently taken an antimalarial dose. This was performed either to complete the treatment course and prevent ‘resistance in the child’ even if there were no detectable parasites or due to expectations of false negative results if an antimalarial was already present in the child's system. Third, a few health workers noted that the RDT detects only *P. falciparum* and a false negative result could occur if the infection is caused by another parasite species.

Provider–client interactions included caregiver RDT mistrust and demand for certain medicines, as well as caregivers’ desires to know the ‘exact’ disease cause if not malaria. Moreover, many caregivers viewed doctors or hospitals as better equipped for differential diagnosis. System constraints included poor referral systems, working by themselves and not able to discuss difficult cases, as well as not having adequate skills or tools to differentiate among other fever causes. Various dilemmas or feelings were reported by respondents about managing these cases in light of these noted system constraints. Health workers expressed doubt about how best to manage these patients as well as feared doing wrong, loss of their patients to follow-up, or even patient death due to malaria. Many also worried that their services would be trusted less by patients, whereas others simply felt unsatisfied for not being able to provide a clear diagnosis to the sick patients.

## Discussion

Overall, the PhD thesis highlighted low and inequitable testing rates for pediatric fevers across multiple sub-Saharan African countries in 2009–2011/12, with significant country differences in the effect of malaria diagnosis on antimalarial drug use patterns at the population level (Studies I and II). Health workers’ perceptions and practices impeded the use of malaria diagnosis for improved pediatric fever management in Uganda and Malawi (Studies III and IV), which needs to be addressed to improve quality fever care and rational use of both antimalarial and antibiotic drugs. Table 6 highlights policy recommendations stemming from the PhD thesis results.

**Table 6:** Policy recommendations

Policy recommendations
Access
• Deploy RDT to communities where pediatric fevers are commonly managed to achieve universal diagnosis goals • Expand integrated community case management to extend access to testing and care in an equitable manner Facility readiness
• Review IMCI guidelines to clarify antibiotic indications in the fever algorithm, particularly for RDT-negative cases
• Integrate RDT and IMCI with combined guidelines, deployment, training, support, and monitoring in order to improve quality fever care and rational use of both antimalarials and antibiotics.
Clinical practice
• Empower health workers at first-level facilities to manage non-severe non-malaria pediatric fevers without referral. This includes, at a minimum, building health worker and community trust in RDT-negative results, reinforcing skills in integrated care, and fostering communities of practice according to the diffusion of innovations theory.

RDT, rapid diagnostic tests; IMCI, Integrated Management of Childhood Illness.

### Access

The PhD thesis found that testing rates were lowest among febrile children who were taken to lower or less formal sources of care where pediatric fevers are commonly managed, particularly by poorest families. This leads to an important socioeconomic dimension of malaria testing found in the PhD thesis, which occurs with other facility-based interventions as well (37). This is consistent with other research showing that the greatest contributor to reduced systems effectiveness for malaria case management is where care is sought for the sick child (38). Indeed, universal diagnosis goals will only be achieved if RDT deployment is targeted to those locations bearing the disproportionate burden of pediatric fever cases that are generally closer to communities. Such targeting would also help reduce significant inequities found in malaria diagnosis, which must be addressed going forward. Integrated community case management with RDT-based malaria diagnosis has been successfully piloted in a number of settings by both community health workers and drug shop owners (39, 40), and it is a promising approach for bringing quality care to remote areas underserved by the formal health system.

### Facility readiness and clinical practice

In both Malawi and Uganda, there was poor integrated pediatric fever management using RDT and IMCI during outpatient consultations for sick children. In Malawi, few sick children were assessed for other fever causes despite a general adherence to RDT results. This included poor assessments of antibiotic need that led to suboptimal antibiotic prescription practices with both over- and undertreatments, which has been described in other settings as well (41, 42). The strong influence of RDT results on antibiotic overtreatment modified by CDB complaints highlights the primary importance of diagnostic test results and patient symptoms on clinical treatment decisions, which has also been described elsewhere (18, 19). These findings may reinforce concerns about growing irrational antibiotic prescription practices in the era of test-based malaria case management (43), particularly given etiology studies from Tanzania showing viral infections cause far more pediatric febrile illnesses than bacterial or parasitic infections (44, 45). As part of recent global commitments to combat antibiotic resistance (46), countries should consider better integration of RDT into IMCI guidelines to improve quality fever care, which could, in turn, improve rational use of both antimalarial and antibiotic drugs. IMCI guidelines should also clarify antibiotic indications in the fever algorithm, particularly for RDT-negative cases, which are currently unclear and could have contributed to antibiotic overtreatment found in this study (47), which has also been demonstrated elsewhere (42).

In Uganda (Mbarara District), health workers reported various challenges or perceptions that drove non-compliance to negative test results, including RDT perceptions, system constraints, and provider–client interactions, which must be addressed in order to improve program performance. Importantly, these stated constraints reflect notable ‘clusters of influence’ on the spread of new innovations or practices according to the diffusion of innovations theory – innovation perceptions, contextual factors, and user characteristics. This theory therefore holds great potential to inform the types of support mechanisms that could help overcome some of the f*acility readiness* and *clinical practices* bottlenecks identified for Mbarara District and may be applicable to other settings.

### Diffusion of innovations – from scientific advancement to effective delivery

The diffusion of innovations theory emerged in the 1960s, with Everett Rogers’ seminal work in rural sociology to understand why, how, and at what rate new innovations spread through a social group (48). Since then, this theory has been used across other disciplines, such as marketing, organizational behavior, and health promotion. There have also been efforts to apply this set of ideas to the uptake of new practices or innovations within health service delivery organizations (48–52). More recently, these ideas were adapted to the RDT experience in sub-Saharan Africa (53), and the current PhD thesis further applied this theory to suggest ways for improving clinical practices in Mbarara District.

According to the diffusion of innovations theory, innovation perceptions play a large role in successful uptake of the new practice or innovation (48–52). Five innovation perceptions may be most important: benefit, compatibility, simplicity, observability, and trialability. In Mbarara District, health workers described the benefits of malaria diagnosis and caregivers were reportedly eager to test their children. Yet, there were also perceived ‘risks’ in missing a malaria case that reduced these perceived *benefit*s. These ‘risks’ should be explicitly acknowledged in training programs and ongoing support mechanisms to improve clinical practices. Similarly, health worker training and tools were not *compatible* with managing RDT-negative cases, and these clients were therefore not perceived as *simple* to manage. *Trialability* and *observability* are also important for adopters in order to have space to test new practices and to understand how others have incorporated these innovations into their work. Some authors further suggest that *trialability* and *observability* of new innovations are especially important for adopting ‘risky’ practices (48, 52).

User characteristics are also important for innovation uptake, and research highlights the central role of ‘opinion leaders’ within a social group to influence the opinion of their peers about the new practice (46–50). Clinical officers could be natural ‘opinion leaders’ among health workers in Mbarara District, but some investment is needed to better connect ‘opinion leaders’ to their professional peers within the district and to further support their role as clinical team leaders, for example, through additional training and financial support. Moreover, evidence points to the importance of fostering informal exchanges among adopters within organizations in order to speed up successful practice adoption (48–52). To this end, recent research found improved RDT adherence practices by facilitating communication among adopters, notably through daily short message service (SMS) reminders or interactive training sessions (54, 55). Other researchers have also proposed initiating Balint groups or peer-learning networks in order to foster *communities of practice* among health workers so that they could regularly discuss and problem-solve clinical practice issues (49, 56). An additional intervention for this district could be providing airtime to nurses for real-time consultations with ‘opinion leaders’ on difficult cases, which could also support such *communities of practice*.

Contextual factors, or system constraints, included weak referral systems, working by themselves and not able to discuss difficult cases, as well as not having adequate skills or tools to differentiate among other fever causes. Indeed, the ultimate objective should be to empower health workers at peripheral clinics to manage non-severe non-malaria fever cases without referral even within the context of a weak health system. As a top priority, RDT should be deployed as part of integrated treatment protocols, notably IMCI for sick children, in order to enhance health workers’ capacity to classify other potential fever causes. As described earlier, *communities of practice* could also potentially help reduce feelings of working alone without support or desires to refer test-negative patients for more advanced testing and care. Nevertheless, some overtreatment of dangerous diseases will – and arguably should – remain the norm in weak health systems, given understandable concerns that a patient may not return if symptoms worsen coupled with difficult referral for more advanced medical care.

### Methodological limitations

There are a number of methodological issues across the four papers, which should be highlighted. First, the PhD thesis primarily relied on secondary analysis of public data sets that does not include in-depth data collection on any one topic. This resulted in missing information needed to help interpret results (e.g. malaria test type) or to construct confounding variables (e.g. illness severity), which is a particular problem in observational studies prone to residual confounding. Secondary analysis of these data sets also resulted in a time lag of a few years between data collection and published results, and our findings therefore reflect practices at the outset of new guidelines. Second, measurement errors may have occurred for various reasons, such as poor recall (e.g. caregiver reporting of test receipt or treatments prescribed) (57, 58) and purposeful misreporting (e.g. provider reporting RDT compliance to please interviewers in qualitative studies). Similarly, providers may also perform better under observation than in routine practice (59). Finally, Study III attempted to identify sick children who should not receive antibiotic prescriptions, which is difficult to measure in settings with inadequate diagnostics. Antibiotic need (or the lack thereof) was based on IMCI pneumonia indications and provider-reported diagnostic categories requiring antibiotics. This definition overestimates antibiotic need (or underestimates ‘without need’) because many children with these classifications or diagnoses will not have the underlying bacterial infection.

## Conclusions

The four studies in this PhD thesis highlight important bottlenecks for using malaria diagnosis for improved pediatric fever management in terms of *access*, *facility readiness*, and *clinical practice* constraints, which must be addressed to improve program performance. RDT deployment should be targeted to community or facility sources of care, where febrile patients are commonly managed, in order to achieve universal diagnosis goals. There is also an urgent need to move beyond malaria-focused *test and treat* strategies toward *IMCI with testing* or *test, classify and treat* in order to improve quality fever care and rational use of both antimalarial and antibiotic drugs in line with recent commitments to combat resistance. Improved overall clinical care, including adherence to malaria test results, also requires stronger health systems, and RDT should be viewed as a unique entry point to support this important effort.

## Paper context

Despite major investments in malaria rapid diagnostic tests (RDTs), small-scale studies from limited countries have indicated poor RDT compliance. This thesis analyzed national surveys from a number of countries to provide large-scale evidence of diagnostic practices including determinants. Malaria testing was low and inequitable with poor RDT integration into clinical guidelines and common RDT mistrust. Going forward, RDT deployment must include integrated treatment protocols, RDT trust-building measures, and health system strengthening efforts to improve overall clinical practices.
